# Supplementary material for: Prospective Longitudinal Changes in the Periodontal Inflamed Surface Area Following Active Periodontal Treatment for Chronic Periodontitis
Source: J Clin Med. 2021 Mar 10;10(6):1165. doi: 10.3390/jcm10061165 (PMC7998532; doi:10.3390/jcm10061165)
Supplement: Supplementary file 1 [file jcm-10-01165-s001.pdf]

Supplemental Material

Changes in the periodontal inflamed surface area during the 24-month follow-up period

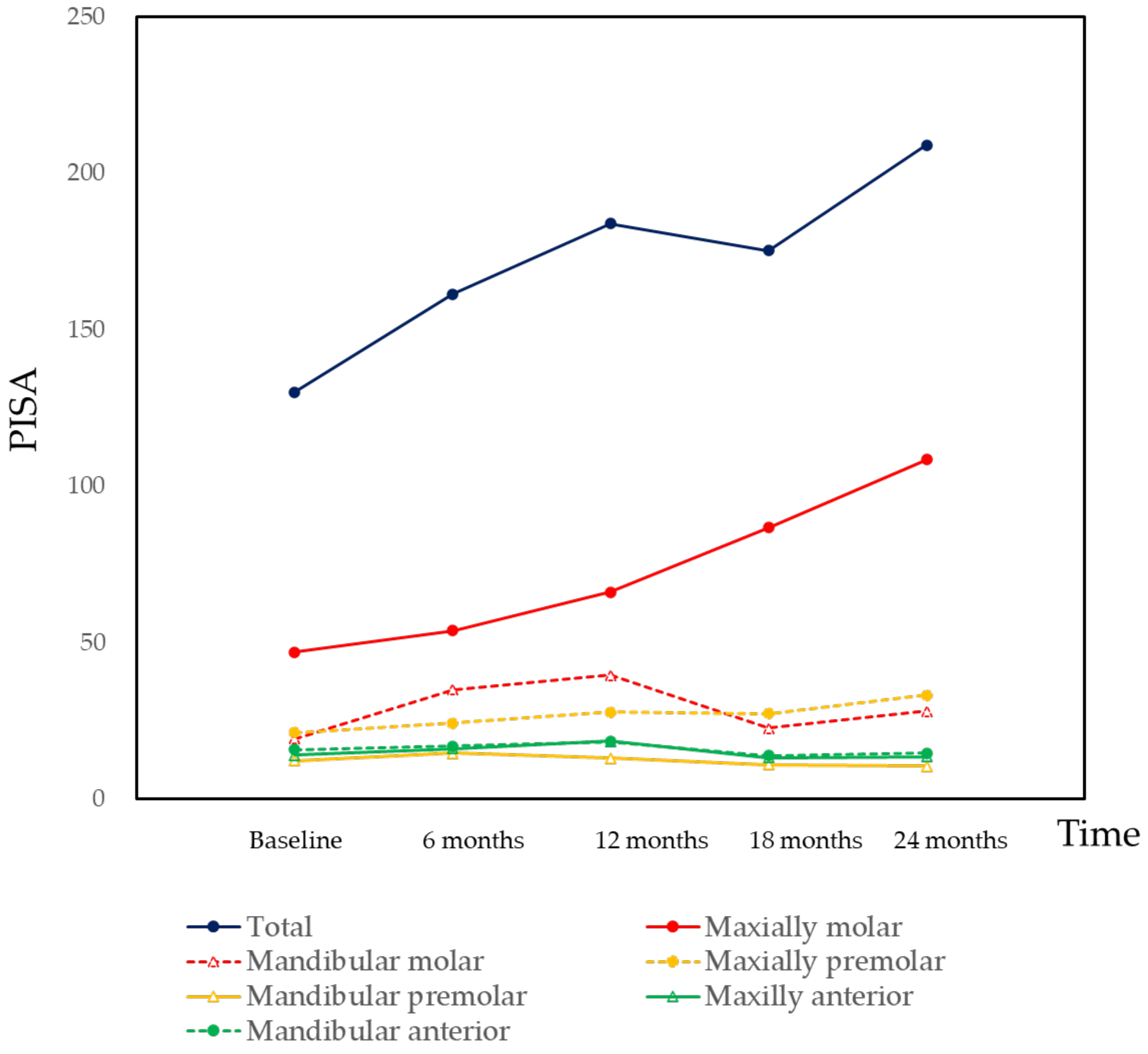

Figure S1. Changes in the periodontal inflamed surface area (PISA) during the 24-month follow-up period according to tooth-type

The PISA of the maxillary molar was increased, which may have affected the total PISA.

Table S1. List of the sample teeth for the evaluation of periodontal pathogens

	Maxillary		Mandibular	
	Right	Left	Right	Left
2nd molar	11	9	4	6
1st molar	18	14	5	2
2nd premolar	7	3	4	1
1st premolar	3	11	2	5
Canine	3	2	1	1
Lateral incisor	4	1	2	0
Central incisor	1	2	2	1

From these sample sites, a paper point was inserted into the periodontal pocket and periodontal pathogens were measured by polymerase chain reaction assay.
